# When immediate losses are followed by delayed gains: Additive hyperboloid discounting models

**DOI:** 10.3758/s13423-019-01599-5

**Published:** 2019-04-22

**Authors:** Sara J. Estle, Leonard Green, Joel Myerson

**Affiliations:** 1 Department of Psychology, University of North Carolina at Greensboro, Greensboro, NC USA; 2 Department of Psychological and Brain Sciences, Washington University in St. Louis, St. Louis, MO USA

**Keywords:** Discounting, Self-control, Choice, Combination outcomes, Immediate losses and delayed gains

## Abstract

Discounting research has tended to focus on one simple situation, choice between an immediate, smaller gain and a larger, delayed gain, that is assumed by many to capture the essence of self-control. In everyday life, however, most choice situations are more complex, often involving combinations of gains and losses. We examined discounting in situations involving an immediate loss followed by a delayed gain that resulted in either a net gain (Experiment 1) or a net loss (Experiment 2) and compared it with discounting when there was only a delayed gain and no immediate loss. Larger delayed gains were discounted less steeply than smaller regardless of whether or not they were preceded by an immediate loss. Discounting functions of the same general hyperboloid form that describe the discounting of delayed gains in simple choice situations accurately described the discounting of combinations of gains and losses, although results differed depending on whether the combination would result in a net gain or a net loss. Participants consistently discounted loss-gain combinations less steeply than gains not preceded by an immediate loss when the combination represented a net loss (Experiment 2), but not when the combination represented a net gain (Experiment 1), a result analogous to the sign effect in simple choice situations (i.e., delayed gains are discounted more steeply than delayed losses). Taken together, these findings support the view that complicated choices like those common in everyday life can be understood within the discounting framework.

## Introduction

The term *delay discounting* refers to the fact that the longer the time until an outcome would occur, the lower its subjective value (i.e., the more its value is discounted). Discounting is believed by many to capture the essence of at least one form of self-control – the ability to delay gratification (Bickel & Marsch, 2001; Odum, 2011). Indeed, groups assumed to lack self-control, such as substance abusers, have been shown repeatedly to discount delayed rewards more steeply than controls (MacKillop et al., 2011).

Discounting is typically studied by having participants choose between smaller, immediate rewards and larger, delayed rewards to determine the amount of immediate reward equal in value to the delayed reward (i.e., its present equivalent). However, such choices represent only one type of situation assumed to require self-control, and few studies have examined the extent to which discounting generalizes to more complicated situations. Such situations are important not only theoretically but also practically because people frequently face such complex choices. Many everyday choices involve an immediate negative consequence paired with a delayed positive outcome. Familiar examples include choosing to buy an item that will not be delivered until a later date, and choosing to attend college, which involves up-front costs (tuition, effort) but is presumed to lead to higher income and more job satisfaction later, as well as many health-related decisions.

Accordingly, the present study examined choices involving an immediate loss to be followed later by a delayed gain. Because participants also made choices involving only delayed gains, we were able to test predictions about how combinations of losses and gains would be discounted. For example, if someone indicated that a $1,200 gain in 6 months had a present, equivalent value of $1,000, a simple additive model predicts they would find the present equivalent of an immediate $900 loss followed in 6 months by a $1,200 gain to be equal to $100 (the sum of the $900 loss and the present equivalent of the delayed $1,200). One previous study (Ostaszewski, 2007) examined choices involving such combinations but did not estimate their present equivalent value, making the results difficult to integrate with the discounting literature.

We conducted two experiments using a titration procedure to estimate the present equivalents of combinations of losses and gains, one in which the combination resulted in a net gain (Experiment 1; e.g., pay $60 now and receive $80 later) and another that was identical except that the combination produced a net loss (Experiment 2; e.g., pay $100 now and receive $80 later). The amounts of both the gains and losses were manipulated in both experiments. When choices are between an immediate and a delayed gain, the steepness with which the delayed gain is discounted decreases systematically with its amount, whereas when choices are between an immediate and a delayed loss, amount has little effect on the degree of discounting (for a review, see Green, Myerson, & Vanderveldt, 2014b). It is not known, however, how amount affects the present equivalent of a delayed gain preceded by an immediate loss, and in particular, given the robust finding of amount effects with delayed gains but not with delayed losses, whether the effect of amount depends on whether the combination of an immediate loss and a delayed gain results in a net gain or a net loss.

Both current experiments examined the validity of a simple additive model in which the combination of an immediate loss and a delayed gain is equal in value to the discounted value of the delayed gain minus the (undiscounted) value of the immediate loss. If true, then as increases in delay decrease the present equivalent value of the future gain, the present equivalent value of the combination of that gain and an immediate loss will become more and more negative. Although intuitively appealing, this idea has not been tested and leaves quite open the question of exactly how the delayed gain component is discounted and the similarities and differences to delay discounting in the absence of a loss component, although the utility of the idea depends on fleshing out these details. The present study addresses such concerns and compares alternative models of decision making in situations where outcomes combine gains and losses that occur at different times.

## Method

### Participants

Experiments 1 and 2 studied 40 (16 males, 24 females; mean age 19.88 years) and 39 undergraduate students (17 males, 22 females; mean age 19.72 years), respectively. These sample sizes greatly exceeded the number needed to provide a power of .80 to detect a difference in the discounting of the smallest and largest delayed gain amounts with alpha equal to .05, as estimated using data from a previous discounting study involving gains and losses (Estle, Green, Myerson, & Holt, 2006). Participants were recruited through the Department of Psychology’s Human Subject Pool and received course credit as compensation.

### Procedure

Participants were tested individually in a small room containing a computer and a monitor, a keyboard, and a mouse. There were two phases to each experiment. In the *gain-only phase* of each experiment, participants chose between a larger delayed amount and a smaller immediate amount; in the *combination phase*, they chose between an immediate loss (payment) followed by a delayed gain, and an immediate amount that could be either a gain or a loss depending on their previous choices. In Experiment 1, the combination phase combined a delayed gain with an immediate loss that was 25% *less* than the gain (e.g., a $60 loss followed by an $80 gain), resulting in a net gain, whereas in Experiment 2, the amount of the loss was 25% *more* than the gain (e.g., a $100 loss followed by an $80 gain), resulting in a net loss. In both experiments, the order of the two phases was counterbalanced across participants. Each phase began with 12 practice trials, after which any procedural questions were answered. Participants were told that the purpose of their experiment was to examine preferences between hypothetical outcomes. They were asked to make their decisions as if the amounts and delays were real and told that there were no correct or incorrect choices.

#### Gain-only phase

The participant made six choices at each of six delays (1 month, 6 months, 1 year, 3 years, 5 years, and 10 years) in three delayed-amount conditions ($80, $1,200, and $20,000). For each participant, the order of the amount conditions was randomly determined, and within each amount condition, each of the delays was presented in a random order before proceeding to the next amount condition. The first choice at each delay was between a delayed gain and an immediate gain equal to half of the delayed gain (e.g., receive $80 in 1 year or receive $40 now). For each of the subsequent choices at that delay, the amount of the immediate gain was adjusted based on the participant’s previous choice, with the size of each adjustment being half of the previous one (see Estle et al., 2006, for examples). If the participant chose the immediate gain, its amount was decreased on the next trial; if they chose the delayed gain, the amount of the immediate gain was increased on the next trial. This iterative procedure rapidly converges upon the amount of an immediate reward approximately equal in value to the delayed gain (i.e., its subjective or present equivalent value plus or minus about 1% of the delayed amount).

#### Combination phase

In this phase, participants were asked to choose between a combination outcome in which they would make a fixed initial payment and receive a larger amount later and a single, immediate outcome. The amount of this immediate outcome was adjusted based on their previous choices as in the gain-only phase in order to estimate the present equivalent of the combination. In Experiment 1, the combinations were a $60 loss and an $80 gain, a $900 loss and a $1,200 gain, and a $15,000 loss and a $20,000 gain; in Experiment 2, the combinations were a $100 loss and an $80 gain, a $1,500 loss and a $1,200 gain, and a $25,000 loss and a $20,000 gain. Notice that the amounts of the delayed gains were the same as those in the gain-only phase and that in Experiment 1, the amounts of the immediate losses were always 75% of the delayed gains with which they were combined, resulting in a net gain, whereas in Experiment 2, the amounts of the immediate losses were always 125% of the delayed gains with which they were combined, resulting in a net loss. The delays were the same as in the gain-only phase. Again, the computer randomly determined the order of both the three delayed amount conditions and the delays.

In both experiments, the alternatives on the first of six choices in each amount and delay condition were an immediate loss followed by a delayed gain, and an immediate outcome whose value was the midpoint between complete discounting of the delayed gain and the absence of discounting of the delayed gain. For example, consider the following combination outcome in Experiment 1: an immediate loss of $60 followed by an $80 gain in 1 year. Complete discounting of the $80 gain would result in its having a present equivalent value of $0; consequently, the value of the combination outcome would be a $60 loss ($60 immediate loss + $0, the present equivalent of the delayed gain). The absence of discounting would mean that an $80 delayed gain is equivalent to an $80 gain now, and thus the net value of the combination outcome would be a $20 gain ($60 immediate loss + $80, the present equivalent value of the delayed gain). The midpoint between these two extremes ($20 and -$60) is -$20, and therefore, participants’ first choices in this condition were between “Pay $20 now” and “Pay $60 now and receive $80 in 1 year.”

For subsequent choices, the amount of the adjusted outcome was increased or decreased based on the participant’s previous choice, following the same algorithm used in the gain-only phase. Using this procedure, the present equivalent of the combination, determined after the sixth choice, could be either positive or negative.

### Analysis

A hyperboloid discounting function (Green, Fry, & Myerson, 1994; Myerson & Green, 1995) was fit to the group mean data from the gain-only phase of both experiments:

1 $$ V=A/{\left(1+ kD\right)}^s, $$

where *V* represents the subjective value of a delayed reward, operationally defined as its present equivalent, *A* represents the amount of the delayed reward, *k* is a parameter governing the rate of discounting, *D* is the delay to its receipt, and *s* represents the nonlinear scaling of amount and time.

According to the simplest additive model, the present equivalent of the combination of an immediate loss followed by a delayed gain is predicted to be the difference between the amount of the loss and the present equivalent of the delayed gain estimated in the gain-only phase. In Experiment 1, for example, a gain of $80 in 1 month was, on average, approximately equal in value to a $69 gain now. Thus, such a simple additive model predicts that the present equivalent of a $60 loss followed in 1 month by an $80 gain is $69 − $60 = $9. A similar transformation was initially used for fitting the hyperboloid discounting function to group mean data from the combination phase. Whereas data from the gain-only phase were fit using Eq. 1, its direct analog, Eq. 2, was used to fit the data from the combination phase:

2 $$ V={A}_{\mathrm{Gain}}/{\left(1+ kD\right)}^s\hbox{--} {A}_{\mathrm{Loss}}. $$

Note that when *D* = 0, *V* = *A*_Gain_*– A*_Loss_ (i.e., the net value of the outcome) and that as *D* approaches infinity, *V* approaches *–A*_Loss_ .

To measure how steeply participants discounted, we calculated the area under their empirical discounting curves (AuCs; Myerson, Green, & Warusawitharana, 2001) as follows. In the gain-only phase, the present equivalents at each delay and amount were converted to relative values (i.e., proportions of the delayed amount). Similarly, in the combination phase, present equivalents were converted to proportions after replacing the amount of the immediate (undiscounted) loss. Thus, continuing with the above example in which the observed present equivalent was $9 and *A*_loss_ was $60, replacing the loss gives a present equivalent value for the gain component of the combination outcome of $69, which was converted to the relative value by expressing it as a proportion of the amount of the delayed gain, (*V+A*_Loss_ ) */ A*_Gain_ = $69/$80 or 0.8625. The areas of the trapezoids formed by these present equivalent values when plotted as a function of delay (expressed as a proportion of the maximum delay) were summed to obtain the AuC. Values may range from 0.0 (complete discounting) to 1.0 (no discounting).

Follow-up analyses were conducted using nonlinear regression to compare different models of any differences in discounting revealed by ANOVAs on the AuCs. The fits of these models were assessed using the proportion of variance accounted for (*R*^2^), the Bayes Information Criterion (BIC), and the root mean square error (RMSE, i.e., the square root of the mean of the squared residuals). Unlike the other measures, the BIC penalizes models based on the number of free parameters so that a model with more parameters that fits as well as or better than a model with fewer parameters might still have a poorer BIC.

## Results

**Fig. 1:**
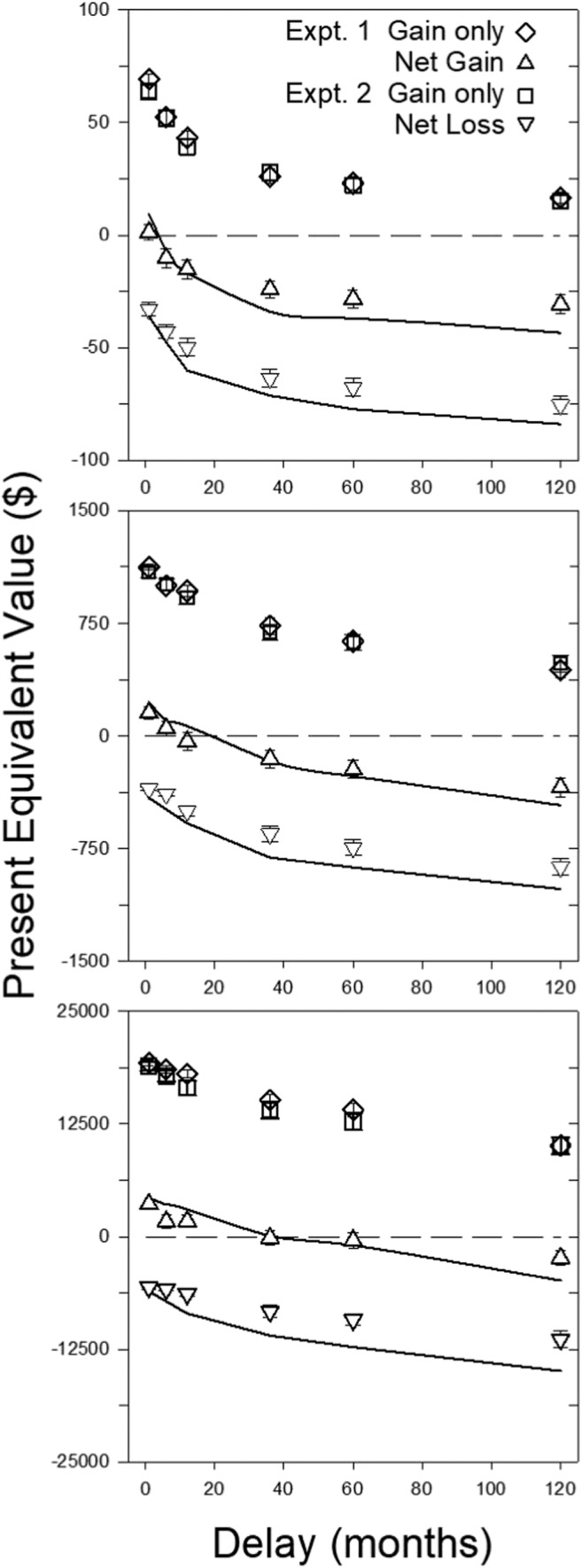
Present equivalent value of a delayed gain or loss-gain combination as a function of delay in Experiments 1 and 2. Symbols represent the group mean present equivalent in each condition; the error bars represent 1 standard error of the mean. The solid lines represent the present equivalents at each delay in the combination phase predicted by subtracting the amount of the immediate loss from the group mean present equivalent at the corresponding delay in the gain-only phase. The data from each amount condition are plotted on a scale ranging between ±125% of the delayed amount in order to facilitate comparison of different amount conditions

Figure 1 presents group mean present equivalent values plotted as a function of delay for both phases of Experiments 1 and 2. As may be seen, present equivalent values declined systematically as a function of delay in all conditions of both phases of both experiments. Notably, the present equivalent of the combination outcomes became increasingly negative as the delay to the gain increased. In fact, the combination phase data showed the same general pattern as the present equivalents predicted by simply subtracting the amount of the immediate loss from the present equivalent value of the corresponding delayed gain in the gain-only phase (solid lines), although they tended to be not as negative as expected at the longer delays.

For both experiments, hyperboloid discounting functions (Eqs. 1 and 2) provided good fits to the group means in all three amount conditions of both the gain-only phase and the combination phase (all *R*^2^s greater than .94), as well as reasonably good fits to the data from individual participants. In both experiments, the median *R*^2^s for the three amount conditions of the gain-only and the combination phases were all greater than .83 and .72, respectively.

**Fig. 2:**
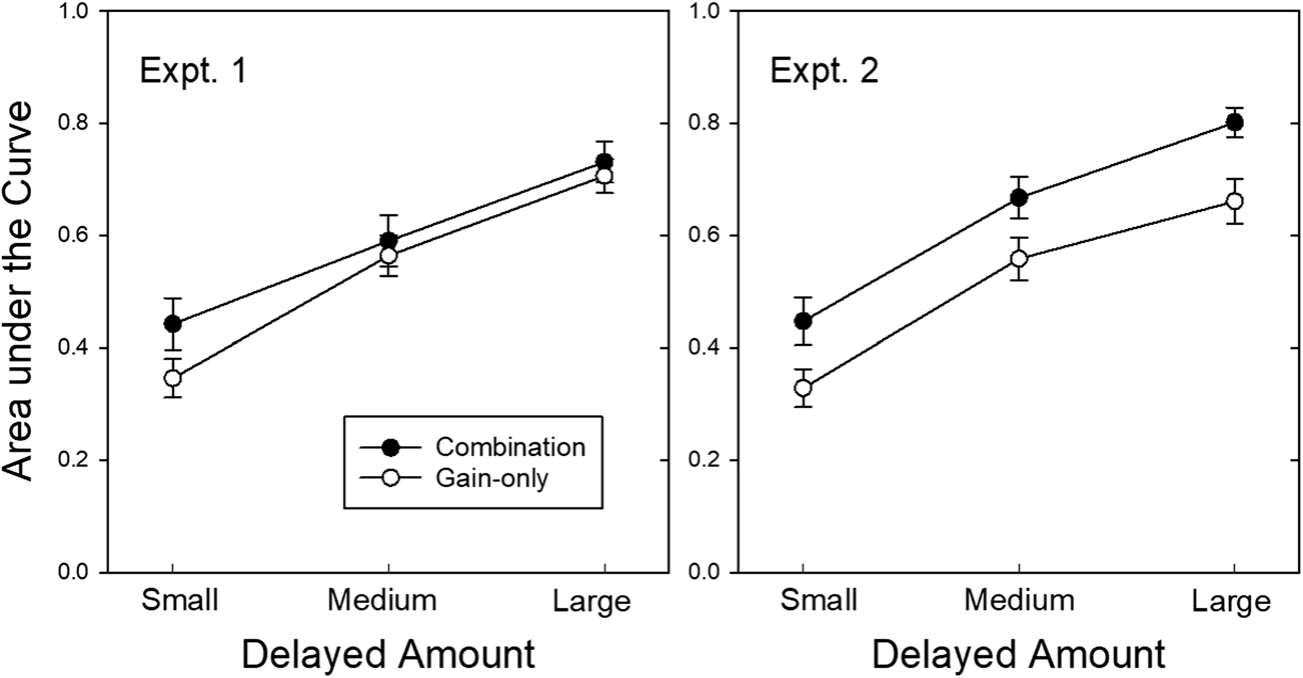
Area under the curve as a function of the amount of the delayed gain in both gain-only and combination phases of Experiment 1 (**left panel**) and Experiment 2 (**right panel**). The error bars indicate the standard error of the mean

AuC measures for each participant in each amount and phase of Experiment 1 were entered into a 2 (Phase) × 3 (Amount) repeated measures ANOVA, which revealed a significant effect of Amount on degree of discounting, *F*(2, 78)=94.64, *p*<.001, η_p_^2^=.708 (see the left panel of Fig. 2). Although the effect of Phase failed to reach significance, *F*(1, 39)=2.88, *p*=.097, these results must be interpreted in light of the significant interaction between Amount and Phase, *F*(2, 78)=5.52, *p*=.044, η_p_^2^=.077. Specifically, the interaction reflected the fact that although the mean AuCs increased with the amount of the delayed gain for both phases and the mean AuCs for the combination phase were always larger than the means for the gain-only phase, this difference was only significant for the smallest ($80) amount, *t*(39)=2.56, *p*=.014. Because of the theoretical significance of the issue, linear contrasts verified that the effect of amount reflected systematic increases in AuC in both phases (i.e., progressively shallower discounting as amount increased), *F*(1,39)=125.77, *p*<.001, η_p_^2^=.763.

Similarly, AuC measures for each participant in each amount condition and phase of Experiment 2 were also entered into a 2 (Phase) × 3 (Amount) repeated measures ANOVA, which revealed a significant effect of phase, *F*(1, 38)=18.67, *p*<.001, η_p_^2^=.329, reflecting the fact that the combination of a delayed gain and an immediate loss was discounted less steeply than the corresponding gain alone. There was also a significant effect of amount, *F*(1, 38)=84.71, *p*<.001, η_p_^2^=.690. The interaction was not significant, *F*(2, 76)<1.0. Again, because of the theoretical significance of the amount effect, a linear contrast was conducted to verify that AuCs increase progressively with the amount of the delayed gain, *F*(1,38)=107.65, *p*<.001, η_p_^2^=.739 (see the right panel of Fig. 2).

Because the distributions of AuCs were not normal in some conditions of Experiments 1 and 2, we conducted six non-parametric follow-up tests comparing AuCs for the gain-only and combination phases, three for each of the two ANOVAs (which should be robust against these deviations) using Mann-Whitney rank sum tests on medians. The results were the same as those obtained with *t*-tests except for the small amount condition of Experiment 1, where no difference was observed. Accordingly, we would emphasize that discounting always differed between the two phases in Experiment 2 regardless of the delayed amount but not in Experiment 1.

The finding that the net loss combinations in Experiment 2 were consistently discounted more shallowly than the corresponding delayed gains, as revealed by the results of the preceding ANOVA, raises the question of whether the difference in degree of discounting reflects a difference in the rate parameter of the hyperboloid model, *k*, or a difference in the exponent, *s*. To address this question, we compared fits of four different models to the group mean data from both phases of each amount condition: a simple additive hyperboloid model (Eq. 2), a model with a separate rate parameter for each phase of the experiment (Eq. 2), a model with a separate exponent for each phase (Eq. 4), and a model with a parameter for scaling the loss (Eq. 5):

3 $$ V={A}_{\mathrm{Gain}}/{\left(1+\left({k}_0+{C}_{\ast }{k}_1\right)\ D\right)}^s\hbox{--} {C}_{\ast }{A}_{\mathrm{Loss}}, $$

4 $$ V={A}_{\mathrm{Gain}}/\left(1+ kD\right){{{{}^{\Big(s}}_0}^{+C}}_{\ast }{{{}^s}_1}^{\Big)}\hbox{--} {C}_{\ast }{A}_{\mathrm{Loss}}, $$

5 $$ V={A}_{\mathrm{Gain}}/{\left(1+ kD\right)}^s\hbox{--} {C}_{\ast }{bA}_{\mathrm{Loss}}, $$

where *C* is a categorical variable distinguishing between the gain-only (*C*=0) and combination phases (*C*=1), the subscripts on the rate parameters and exponents indicate the corresponding phase (gain-only = 0; combination = 1; no subscript indicates same for both phases), and *b* is a free parameter that scales the immediate loss.

The fit statistics for Eqs. 2, 3, 4, and 5 from each amount condition are presented in Table 1. Comparing the fits to the data from the gain-only and combination phases of the three net gain conditions (Experiment 1), it may be seen that including separate exponents for the gain-only and combination phases resulted in the best model of the four (i.e., Eq. 4 was the model with the most negative BIC) when the delayed gain was small, but adding an additional parameter, either *k*, *s*, or *b* (Eqs. 3, 4, and 5, respectively), did not improve the fits over the simple additive model (Eq. 2) for the data from the other two amounts. These results are consistent with the results of the ANOVA on the AuCs from this experiment, which revealed a significant amount × phase interaction, reflecting the fact that the difference between the AuCs for the gain-only and combination phases was only significant when the delayed gain was small. Importantly, these results localize the source of the significantly shallower discounting in the combination phase, revealing that it reflects a lower value of the exponent, *s*, for that phase, not a difference in *k*.

**Table 1:** Fit statistics (*R*^2^, BIC, and RMSE) for different discounting models (Eqs. 2, 3, 4, and 5) fitted to the data from the gain-only and combination phases of Experiment 1 (Net Gain) and Experiment 2 (Net Loss)

	Eq. 2 (1*k*,1*s*)	Eq. 3 (2*k*,1*s*)	Eq. 4 (1*k*,2*s*)	Eq. 5 (1*k*,1*s*,1*b*)
Net Gain				
Small				
*R* ^2^	.981	.983	.988	.984
BIC	-151.79	-152.15	-164.86	-154.09
RMSE	4.38	4.16	3.56	4.07
%RMSE	5.5	5.2	4.4	5.1
Medium				
*R* ^2^	.990	.990	.990	.990
BIC	-174.88	-171.19	-172.35	-173.54
RMSE	51.09	51.08	50.23	49.60
%RMSE	4.3	4.3	4.2	4.1
Large				
*R* ^2^	.987	.987	.988	.988
BIC	-166.95	-163.27	-165.52	-165.85
RMSE	909.00	907.45	882.57	1016.94
%RMSE	4.5	4.5	4.4	4.4
Net Loss				
Small				
*R* ^2^	.990	.997	.997	.995
BIC	-170.74	-209.55	-215.57	-194.87
RMSE	4.97	2.90	2.65	3.48
%RMSE	6.2	3.6	3.3	4.4
Medium				
*R* ^2^	.992	.999	.999	.997
BIC	-170.74	-251.30	-245.29	-211.85
RMSE	63.22	24.93	27.14	41.48
%RMSE	5.3	2.1	2.3	3.5
Large				
*R* ^2^	.989	.999	.999	.995
BIC	-167.16	-272.32	-258.41	-197.24
RMSE	1248.51	308.32	371.09	809.35
%RMSE	6.2	1.5	1.9	4.0

*Note: R*^2^ represents the proportion of variance accounted

*BIC* represents Bayes Information Criterion, *RMSE* root mean square error (i.e., the square root of the mean squared residual), *%RMSE* the RMSE as a percentage of the delayed amount

Note that for the BIC, a more negative value indicates a better fit

In contrast, when the combination of the immediate loss and delayed gain represented a net loss (Experiment 2), a simple additive model (Eq. 2) with only two free parameters consistently resulted in the poorest fits to the data. Of the three-parameter models, the model with separate *k*s (Eq. 3) and the model with separate exponents (Eq. 4) for the gain-only and combination phases consistently resulted in more negative BICs than the model with a free parameter that scaled the immediate loss (Eq. 5). However, neither of the other two models (Eq. 3 and Eq. 4) was consistently the best. Thus, the consistently shallower discounting in the combination phase, regardless of the amount of delayed gain, could reflect lower values of *k* and/or the exponent. Despite the observed differences in the fits of the different discounting models (Eqs. 2–5), it is to be noted that they all provided excellent fits, as indicated by *R*^2^s greater than .980.

## Discussion

The present study reports results from a new experimental paradigm for studying choices in which, as in many everyday choice situations, there is an immediate loss followed later by a delayed gain – a situation often characterized as involving self-control. The study’s major goal was to explore the idea that the present equivalent value of an immediate loss followed by a delayed gain equals the present equivalent of the gain minus the amount of the loss. Research on discounting has, with few exceptions, focused on choices between unitary outcomes, with the iconic situation involving a choice between a smaller, immediate financial gain and a larger, delayed financial gain. Although a tendency toward steep discounting is frequently interpreted as reflecting a general impulsivity trait that manifests itself in a wide variety of situations calling for self-control, it has been unclear whether the discounting framework can be applied to decisions in situations where at least one of the choice alternatives leads to a combination of outcomes.

The present results are generally consistent with the idea that value is additive in combination outcomes, leading to the correct prediction that as increases in delay decrease the present equivalent value of a future gain, the present equivalent value of the combination of that gain and an immediate loss will become more and more negative. Importantly, this pattern was observed regardless of the amounts of the losses and gains involved, and regardless of whether the combination represented a net gain (Experiment 1) or a net loss (Experiment 2). Although the present approach is reminiscent of the additive-utility theory proposed by Killeen (2009), that theory focuses on attributes of a single event (e.g., the delay until a monetary reward of a specific amount). In contrast, the present approach concerns combinations of separate events (i.e., a loss followed by a delayed gain) that, together, are the outcome of a person’s choice. Such combinations have received little attention in the literature despite the fact that they are part of many everyday choice situations.

One important aspect of the current study concerns the effect of amount on the present value of combination outcomes. Amount effects, in which discounting decreases with the amount of the delayed gain, are almost always observed when choice outcomes only involve gains (Green, Myerson, Oliveira, & Chang, 2013), but not when the outcomes only involve losses (Estle et al., 2006; Green, Myerson, Oliveira, & Chang, 2014a), raising the question of what happens to amount effects when gains and losses are combined. An answer to this question is provided by the significant effects of amount in both experiments. That is, the combination of an immediate loss and a delayed gain behaved like a gain in that shallower discounting of larger gains was observed regardless of whether the combination resulted in a net gain or a net loss.

At the same time, however, the combination tended to behave either like a gain or a loss, depending on whether the net value of the combination (undiscounted gain – loss) was positive or negative. Specifically, combinations resulting in net losses tended to behave like simple delayed losses in that they were discounted less steeply than the delayed gain alone, a result analogous to the well-known sign effect with simple delayed outcomes (Thaler, 1981). In contrast, combinations resulting in net gains were discounted at the same rate as the gain alone for two of the three delayed amounts studied.

One notable aspect of discounting with outcomes that are not combinations (e.g., delayed gains or losses, probabilistic gains or losses) is how well it is described by a simple two-parameter hyperboloid model (Green, Myerson, & Vanderveldt, 2014b; Myerson & Green, 1995), and thus it was of interest whether such a model could be adapted to describe the discounting of combination outcomes. Although the simplest hyperboloid model (Eq. 2) provided good descriptions of participants’ choices in all conditions, when the combination outcome represented a small net gain or a net loss of any amount, adding a third free parameter resulted in a better fit, while remaining consistent with the fundamental idea that the present equivalents of combination outcomes represent the present equivalent of the delayed gain (i.e., its discounted value) minus the immediate cost.

Only in the case of the small net gain was it possible to choose between a model with two discounting rate parameters and a model with two exponents, with the latter providing a better fit. In the case of net losses, adding either a separate rate parameter or exponent improved the fit approximately equally, leaving open the question of what underlies the shallower discounting of combination outcomes in such situations. In fact, previous studies comparing gains and losses suggest not only that both the rate parameter and the exponent will be different when the combination represents a net loss, but also that losses are scaled differently than gains (e.g., Estle et al., 2006; Kahneman & Tversky, 1979). Thus, the best model could be one that represents a combination of Eqs. 3, 4, and 5. It should be noted, however, that fits of the current models resulted in *R*^2^s very close to ceiling. Thus, an approach based solely on comparing fit statistics may not be productive here, and alternative approaches to evaluating alternative models may be needed. Nevertheless, the fact that in the case of net losses, adding a third free parameter consistently improved the fit as measured by the BIC, which penalizes for additional parameters, indicates that such parameters are justified and demonstrates that an additive model can provide an accurate description of the data regardless of whether a combination outcome is discounted to the same degree as a single gain or not.

Although a wide range of amounts were studied, the combination outcomes were limited in that losses were always 25% less or 25% more than the amount of the delayed gain. Future research will need to examine combinations where losses are smaller and larger percentages of the delayed gain as well as with combinations of immediate gains and delayed losses, the opposite of the situation examined here. Research also is needed to determine if similar results are observed with combinations involving qualitatively different positive and negative outcomes.

A broader examination of choice situations is needed because the situations in which people find themselves are typically both more complicated and more varied than those studied to date. Simply assuming that a single discounting process or trait underlies decision making in all cases seems overly optimistic. Still, we are encouraged by the current findings that show the broad applicability of the discounting framework, even if the way it is best applied varies from one type of situation to another. Clearly, there is much to be learned from exploration of choice situations that, like those frequently encountered outside the laboratory, are more complicated than those usually studied in the laboratory.

### Author Note

The research was supported by National Institutes of Health Grant MH055308; preparation of the manuscript was supported in part by the National Institute on Aging of the National Institutes of Health under Award Number R01AG058885. The research is based on the dissertation of SJE in partial fulfillment of the requirements for the doctoral degree in Psychology from Washington University. We thank members of the Psychonomy Cabal for their help in data collection and Yu-Hua Yeh for his suggestions and statistical assistance.
